# Induction of immune tolerance and reduction of aggravated lung eosinophilia by co-exposure to Asian sand dust and ovalbumin for 14 weeks in mice

**DOI:** 10.1186/1710-1492-9-19

**Published:** 2013-06-03

**Authors:** Miao He, Takamichi Ichinose, Seiichi Yoshida, Hirohisa Takano, Masataka Nishikawa, Guifan Sun, Takayuki Shibamoto

**Affiliations:** 1 Department of Health Sciences, Oita University of Nursing and Health Sciences, 870-1201 Oita, Japan; 2 Department of Environmental Engineering, Environmental Health Division, Graduate School of Engineering, Kyoto University, Kyoto daigaku-Katsura, Nishikyo-ku, Kyoto 615-8530, Japan; 3 Environmental Chemistry Division, National Institute for Environmental Studies, 305-8506 Tsukuba, Ibaraki, Japan; 4 Department of Environmental and Occupational Health, College of Public Health, China Medical University, 11001 Shenyang, China; 5 Department of Environmental Toxicology, Takayuki Shibamoto, University of California, Davis, CA 95616,, USA

## Abstract

**Background:**

Atmospheric contamination caused by Asian sand-dust (ASD) storms aggravates asthma in both human adults and children. This study aims to investigate a series of manifestations in allergic airway disease caused by co-exposure to allergens and ASD for 6 weeks and 14 weeks.

**Methods:**

CD-1 Mice were instilled intratracheally with 0.1 mg of ASD/mouse four times (6 weeks) or eight times (14 weeks) at 2-week intervals (total dose of 0.4 mg or 0.8 mg/mouse) with or without ovalbumin (OVA). The pathologic changes in the airway, cytological alteration in bronchoalveolar lavage fluid (BALF), and levels of inflammatory cytokines/chemokines in BALF, and OVA-specific IgE and IgG1 antibodies in serum were measured in the treated CD-1 mice.

**Results:**

Four-time co-exposure to OVA and ASD aggravates allergic airway inflammation along with Th2-cytokine IL-13 and eosinophil-relevant cytokine/chemokines IL-5, Eotaxin and MCP-3 in BALF, and fibrous thickening of the subepithelial layer in the airway. On the other hand, eight-time co-exposure attenuates these changes along with a significant increase of TGF-β1 in BALF. Adjuvant effects of ASD toward IgG1 and IgE production in sera were, however, still seen in the eight-time co-exposure.

**Conclusions:**

These results indicate that the immune responses in airways are exacerbated by four-time co-exposure to ASD with OVA, but that there is a shift to suppressive responses in eight-time co-exposure, suggesting that the responses are caused by TGF-β1-related immune tolerance.

## Background

Asian sand dust (ASD) storms arise annually from the Gobi Desert, the Taklimakan desert, and loess areas of interior China during the spring season and/or sometimes during the autumn season every year [1]. ASD aerosol spreads through downwind areas, such as East China, the Korean Peninsula, and Japan as well as across the Pacific Ocean to the United States [2-4]. It is also reportedly that ASD transported one full circuit around the globe [5]. Moreover, recent researches point out that the frequency of ASD storm increases rapidly after the year of 2000, and ASD storm may enter a new active period [6].

A major public concern on ASD is its potential hazardous-effect toward respiratory diseases in the Eastern Asian countries. ASD aerosol contains various toxic materials, including by-product materials derived from combustion of a fossil fuel like polycyclic aromatic hydrocarbons (PAHs), sulfate (SO_4_^2−^), and nitrate (NO_3_^−^) and microbial agents, such as bacteria, fungi, fungal spores, and viruses [7-9]. ASD is also known to be composed of 60% silica [10].

Results of epidemiologic studies have shown that ASD caused an increase in hospitalization for pneumonia in China [11], an increase of acute respiratory symptoms in child asthma [12], deterioration of pulmonary function of asthmatic patients and aggravation of their symptoms at night in Korea [13], and an increase in daily admissions and clinic visits for asthma [14] in Taiwan. In Japan, there are reports on the exacerbation of Japanese cedar pollinosis and seasonal allergic rhinitis [15] as well as of adult asthma [16] occurring during a dust storm event. In Toyama, Japan, heavy ASD events also increase hospitalization of children ages 1–15 due to asthma attacks [17].

Previously we reported ASD enhanced *Klebsiella pneumonia* lung inflammation [18], and aggravated OVA associate-lung eosinophilia in the case of four-time treatment of OVA + ASD used in healthy mice [10]. In a recent study, we demonstrated that a one-time treatment of ASD has a potent effect in activating lung eosinophilia in mice immunized beforehand by OVA [19].

It is important to investigate a series of manifestations in allergic airway disease caused by eight-time exposure to allergen and ASD when devising a clinical strategy for dealing with ASD-stimulated allergic airway disease. However, there are no experimental studies on the effects of eight-time exposure to ASD on lung eosinophilia.

Asian dust event with the ASD aerosol intermittently occur during mid-February ~ May (14 weeks) in the spring season. In the present study, two time-course studies (6 weeks and 14 weeks) were set to investigate a series of manifestations in lung eosinophilia caused by intratracheal co-exposure to ASD and ovalbumin. The pathologic changes in the airway, cytological alteration in bronchoalveolar lavage fluid (BALF), and levels of inflammatory cytokines/chemokines in BALF, and OVA-specific IgE and IgG1 antibodies in serum were investigated in CD-1 mice.

## Materials and methods

### Animals

Male CD-1 mice (5 weeks of age) were purchased from Charles River Japan, Inc. (Kanagawa, Japan). Abnormal body weight and sick mice were examined for one week and removed from the pool of subjects. The remaining healthy mice (128 mice) were used at 6 weeks of age. Mice were fed a commercial diet CE-2 (CLEA Japan, Inc., Tokyo, Japan) and given water *ad libitum*. Mice were housed in plastic cages lined with soft wood chips. The cages were placed in a room air conditioned at 23°C and 55–70% humidity with a light/dark (12 h/12 h) cycle. CD-1 male mice were used because of their moderate responsiveness to airway inflammation caused by OVA or mite allergen treatment [20]. The study adhered to the U.S. National Institutes of Health Guidelines for the use of experimental animals. The animal care method was approved by the Animal Care and Use Committee at Oita University of Nursing and Health Sciences in Oita, Japan.

### Asian sand dust particle

The present study used ASD previously collected from Iki Island, Japan on March 21^st^ to 22nd, 2002 after a massive 3-day dust storm event occurred in East Asia. As previously reported, the chemical composition of this ASD sample was 61.8% SiO_2_, 13.6% Al_2_O_3_, 5.7% Fe_2_O_3_, 5.4% CaO, 3.3% MgO, 0.01% TiO_2_, and 2.6% K_2_O. The size distribution peak of ASD was observed at 4.7 μm. The concentration of SO_4_^2−^, NO_3_^−^, and Cl^−^ was 15,000 pg/g, 5000 pg/g, and 7000 pg/g. The concentration of lipopolysaccharide (LPS) and β-glucan was 1.06 EU/mg and 76 pg/mg, respectively [10].

### Study protocol

CD-1 mice were divided into eight groups (n = 16, each group) according to the treatment with particles: as the 6-week study, (1) Control (4-Control): intratracheal instillation with 0.1 ml of normal saline per mouse four times at 2-week intervals; (2) ASD (4-ASD): intratracheal instillation with ASD four times at 2-week intervals; (3) OVA (4-OVA): intratracheal treatment with OVA four times at 2-week intervals; (4) OVA + ASD (4-O+A): intratracheal treatment with OVA and ASD four times at 2-week intervals; as the 14-week study, (5) Control (8-Control): intratracheal instillation with 0.1 ml of normal saline per mouse eight times at 2-week intervals; (6) ASD (8-ASD): intratracheal instillation with ASD eight times at 2-week intervals; (7) OVA (8-OVA): intratracheal treatment with OVA eight times at 2-week intervals; (8) OVA + ASD (8-O+A): intratracheal treatment with OVA and ASD eight times at 2-week intervals. The ASD particles were suspended in normal saline (0.9% NaCl) for instillation (Otsuka Co, Kyoto, Japan). This suspension was sonicated for 5 min with an ultrasonic disrupter, UD-201 type with micro tip (Tomy, Tokyo, Japan), under cooling conditions. The instillation volume of the suspension was 0.1 ml/mouse. The one time instillation dose of ASD was 0.1 mg /mouse. Therefore, the total administration doses of ASD were 0.4 mg/mouse and 0.8 mg/mouse, respectively. A massive Asian dust storm event occurred in East Asia from March 20–22, 2002. The average density of the ambient particulate matter or TSP (total number of suspended particles/m^3^) was 672 μg – 796 μg/m^3^/day in Iki-island, Nagasaki, Japan and 10 mg/m^3^/day in Beijing [21]. A brief description of the instillation dosing of ASD is as follows: When the body weight of the mice is reached about 36 g, the tidal air volume was approximately 0.15 mL and the breathing rate was about 200 breaths/min. The amount of ASD deposited in the lungs of a single mouse per day (2.16 μg) was calculated using the inhaled value for suspended particulate matter (SPM) of 0.1 mg/m^3^, as set by the Japanese national air quality standard (JNAQS).

If 100% of 10 mg/m^3^/day - which is the concentration in previous record of Beijing - is accumulated in a lung (216 μg/day), the one time instillation dose (100 μg) of particles used in the present study would be the equivalent of 0.46 times the amount accumulated in a lung. In the case of the Human Respiratory Tract Model for Radiological Protection [22], the deposition rate into alveoli is approximately 3% for a 6 μm diameter particle. The amount of 3% deposition of 216 μg/day is approximately 6.48 μg/day. In the case of 6 weeks or 14 weeks of exposure at 10 mg/m^3^/day, the accumulated amount is 546 μg or 1274 μg, respectively. The total instillation dose (400 μg or 800 μg) of particles used in the present study would, therefore, be 0.73 times or 0.62 times the amount accumulated in a lung. The atmospheric concentration of 6~10 mg/m^3^/day during the spring season sometimes occur in China. Therefore, we used a 0.1 mg dose of ASD.

OVA was dissolved in the same saline. The one time treatment dose of OVA was 1 μg /mouse (total does of 4 μg or 8 μg /mouse). Mice were intratracheally instilled with these particles through a polyethylene tube under anesthesia with 4% halothane (Takeda Chemical, Osaka, Japan). One day after the last intratracheal administration, the mice from all groups (age = 12 weeks and age=20 weeks) were killed by exsanguination under deep anesthesia by intraperitoneal injection of pentobarbital.

### Pathological evaluation

Eight of the 16 mice from each group were used for a pathologic examination. The lungs were fixed by 10% neutral phosphate-buffered formalin. After separation of the lobes, 2 mm thick blocks were taken for paraffin embedding. Embedded blocks were sectioned at a thickness of 3 μm, and then were stained with hematoxylin and eosin (H&E) to evaluate the degree of infiltration of eosinophils or lymphocytes in the airway from proximal to distal. The sections were also stained with periodic acid–Schiff (PAS) to evaluate the degree of proliferation of goblet cells in the bronchial epithelium. A pathological analysis of the inflammatory cells and epithelial cells in the airway of each lung lobe on the slides was performed using a Nikon ECLIPSE light microscope (Nikon Co, Tokyo, Japan). The degree of proliferation of goblet cells in the bronchial epithelium was graded on the following scale: 0, not present; 1, slight; 2, mild; 3, moderate; 4, moderate to marked; and 5, marked. ‘Slight’ was defined as less than 20% of the airway with goblet cells stained with PAS; ‘mild’ as 21–40%; ‘moderate’ as 41–60%; ‘moderate to marked’ as 61–80%; and ‘marked’ as more than 81% [10]. The degree of thickening of the subepithelial layer in the main bronchus was graded on the following scale: 0, not present; 1, slight; 2, mild; 3, moderate; 4, moderate to marked; and 5, marked. ‘Slight’ was defined as 5–12 μm of the main bronchus with fibroblasts stained with PAS; ‘mild’ as 13–20 μm; ‘moderate’ as 21–28 μm; ‘moderate to marked’ as 29–36 μm; and ‘marked’ as more than 37 μm. Pathological changes were assessed on one slide stained with PAS per mouse. This evaluation procedure was performed by two pathologists who cross-checked the data in blinded specimens. All values were expressed as mean ± SD (n = 8).

### Bronchoalveolar lavage fluid (BALF)

The remaining eight mice from each group were used for an examination of the free cell contents from BALF. BALF and cell counts were conducted using a previously reported method [10]. In brief, the tracheas were cannulated after the collection of blood. The lungs were lavaged with two injections of 0.8 ml of sterile saline at 37°C by syringe. The lavaged fluid was harvested by gentle aspiration. The average volume retrieved was 90% of the amount instilled (1.6 ml). The fluids from the two lavages were combined, cooled to 4°C, and centrifuged at 1500 rpm for 10 min. The total amount of lavages collected from individual mice was used in order to the measure the protein levels of cytokines and chemokines in the BALF. The total cell count of a fresh fluid specimen was determined using a hemocytometer. Differential cell counts were assessed on cytologic preparations. Slides were prepared using a Cytospin (Sakura Co, Ltd, Tokyo, Japan) and stained with Diff-Quik (International Reagents Co, Kobe, Japan). A total of 300 cells were counted under oil immersion microscopy. The BALF supernatants were stored at −80°C until analyzed for cytokines and chemokines.

### Quantitation of cytokines and chemokines in BALF

The cytokine protein levels in the BALF were determined using enzyme-linked immunosorbent assays (ELISA). Interleukin (IL)-1β, IL-4, IL-6, IL-10, IL-13, IL-17A interferon (IFN)-γ, eotaxin, keratinocyte chemoattractant (KC), monocyte chemotactic protein (MCP)-1, macrophage inflammatory protein (MIP)-1α, RANTES and tumor necrosis factor (TNF)-α, as fibrogenic parameters, fibroblast growth factor (FGF)-2, platelet-derived growth factor (PDGF)-BB, transforming growth factor (TGF)-β1 were measured using an ELISA kit from R&D Systems Inc. (Minneapolis, MN, USA). IL-5, IL-12 were measured using an ELISA kit from Endogen (Cambridge, MA, USA). MCP-3 was measured using an ELISA kit from Bender MedSystems (Burlingame, CA, USA).

### Antigen-specific IgE and IgG1 antibodies

OVA-specific IgE and IgG1 antibodies were measured using a Mouse OVA-IgE ELISA kit and a Mouse OVA-IgG1 ELISA kit (Shibayagi Co, Shibukawa, Japan). According to the manufacturer’s protocol, 1U of the anti-OVA IgE is defined as 1.3 ng of the antibody, and 1U of the anti-OVA IgG1 as 160 ng of the antibody. The absorption at 450 nm (sub-wave length, 620 nm) for OVA-specific IgE and IgG1antibodies was measured using a microplate reader (Spectrafluor, Tecan, Salzburg, Austria).

### Statistical analysis

Statistical analyses of the pathologic evaluation in the airway, cytokine and chemokine proteins in BALF were conducted using the Tukey Test for Pairwise Comparisons (KyPlot Ver.5, Kyens Lab Inc, Tokyo, Japan). Differences among groups were determined as statistically significant at a level of p < 0.05.

## Results

### Enhancement of cell numbers in BALF by ASD

**Figure 1:**
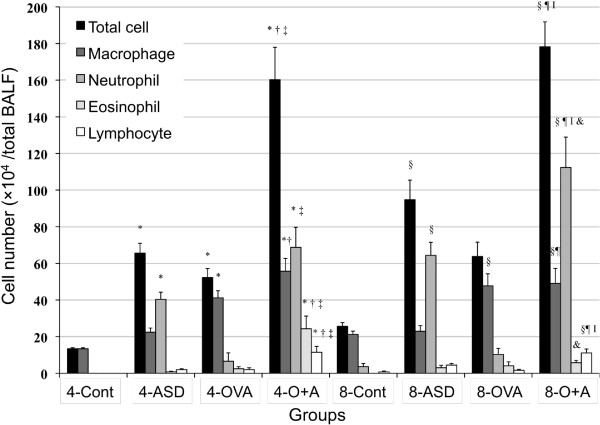
**Cellular profile in bronchoalveolar lavage fluid (BALF).** All values expressed as mean ± *SE*. ^*^p < 0.001 *vs* 4-Control, ^†^p < 0.01 *vs* 4-Control, ^‡^p < 0.05 *vs* 4-Control, ^§^p < 0.001 *vs* 4-ASD, ^¶^p < 0.001 *vs* 4-OVA, ^I^p < 0.001 *vs* 8-Control,^**^p < 0.01 *vs* 8- Control, ^††^*p* < 0.001 *vs* 8- ASD, ^‡‡^*p* < 0.01 *vs* 8-ASD, ^§§^*p* < 0.05 *vs* 8-ASD, ^¶¶^*p* < 0.001 *vs* 8-OVA, ^II^*p* < 0.001 *vs* 4-O+A, ^†††^*p* < 0.01 *vs*. 4-O+A, &*p* < 0.05 *vs* 4-O+A.

To estimate the effects of four-time and eight-time exposure to ASD on the magnitude of airway inflammation caused by OVA, the cellular profile of BALF was examined and results are shown in Figure 1. Both four-time and eight-time exposure to ASD alone increased the number of total cell (four-time, *p* < 0.01; eight-time, *p* < 0.001) and neutrophil (four-time, *p* < 0.05; eight-time, *p* < 0.001) compared with their respective controls. Four-time co-exposure to ASD and OVA strongly increased the number of total cell, eosinophil, and lymphocyte compared with the four-time control, ASD alone, and OVA alone (*p* < 0.001).

Eight-time co-exposure to ASD and OVA significantly enhanced the number of total cell (*p* < 0.001), neutrophil (Control, *p* < 0.001; ASD, *p* < 0.01; OVA, *p* < 0.001) and lymphocyte (Control, *p* < 0.001; ASD, *p* < 0.05; OVA, *p* < 0.001) compared with the 8-control, ASD alone, and OVA alone. Furthermore, eight-time co-exposure showed a remarkable increase in the number of neutrophil compared with the four-time co-exposure (*p* < 0.01). However, the number of eosinophil in the eight-time co-exposure to ASD and OVA group was markedly less than that of the four-time co-exposure group (*p* < 0.001). In addition, both four-time (*p* < 0.001) and eight-time (*p* < 0.01) co-exposure to ASD and OVA elevated the number of macrophage compared with the control and ASD alone.

### Enhancement of pathologic changes in the airway by ASD

**Figure 2:**
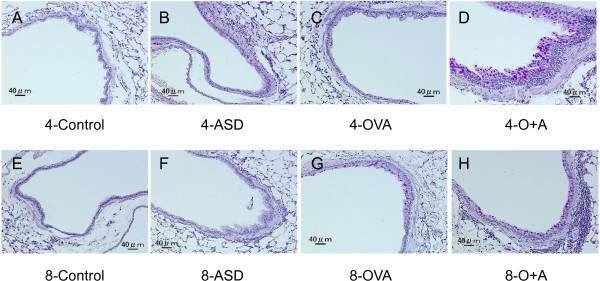
**Effects of ASD on goblet cells and subepithelial layer of the airway.** (**A**) No pathological changes in lungs treated with saline for four times. (**B**) Slight bronchitis in the airway exposed to ASD alone for four times. (**C**) Very slight proliferation of goblet cells in the airway epithelium exposed to OVA alone for four times. (**D**) Marked proliferation of goblet cells that have mucus stained pink with PAS solution, slight infiltration of inflammatory cells into connective tissue around the airway and marked fibrous thickening of the subepithelial layer in the main bronchus treated with OVA and ASD for four times. (**E**) No pathological changes in lungs treated with saline for eight times. (**F**) Slight proliferation of bronchial cells and bronchitis in the airway exposed to ASD alone for eight times. (**G**) Mild proliferation of goblet cells in the airway epithelium exposed to OVA alone for eight times. (**H**) Mild proliferation of goblet cells, moderate fibrous thickening of the subepithelial layer and moderate infiltration of inflammatory cells into connective tissue around the airway co-exposed to OVA and ASD for eight times. (**A–H)** PAS stain.

**Figure 3:**
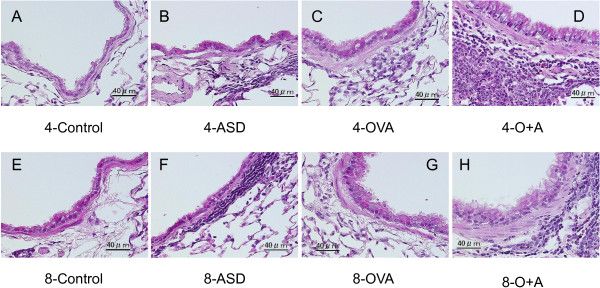
**Effects of ASD on infiltration of inflammatory cells in the airway.** (**A**) No pathological changes treated with saline for four times. (**B**) Slight hypertrophy of epithelial cells and slight infiltration of neutrophils and lymphocytes in the airway exposed to ASD alone for four times. (**C**) Slight proliferation of epithelial cells and very slight infiltration of eosinophils and lymphocytes into the airway submucosa and exposed to OVA alone for four times. (**D**) Marked infiltration of eosinophils and lymphocytes into the airway submucosa, extension of length of epithelial cells in the airway epithelium co-exposed to OVA and ASD for four times. (**E**) No pathological changes treated with saline for eight times. (**F**) Slight infiltration of lymphosytes in the airway exposed to ASD alone for eight times. (**G**) Very slight infiltration of eosinophils into the airway submucosa and proliferation of epithelial cells in the airway exposed to OVA alone for eight times. (**H**) Moderate infiltration of eosinophils and lymphocytes into the airway submucosa, proliferation of epithelial cells co-exposed to OVA and ASD for eight times. (**A–H**) H&E stain.

To determine the effects of four-time and eight-time exposure to ASD on lung pathology-related exposure, the lung specimens stained with HE and PAS were evaluated. Table 1 shows the pathologic changes caused by the ASD in the murine airway, and Figure 2 (PAS stain) and 3 (HE stain) illustrate the effects of the ASD on pathological changes in the lungs. No pathologic alterations were found in the lungs of the four-time and eight-time control (Figures 2A, 2E, 3A, and 3E). The four-time exposure to ASD alone caused slight bronchitis along with proliferation of bronchial cells and alveolitis with neutrophilic inflammation (Figures 2B and 3B). The eight-time exposure to ASD alone resulted in moderate bronchitis along with proliferation of bronchial cells and slight thickening beneath the basement membrane and infiltration of lymphocytes and neutrophils (Figures 2F and 3F). The four-time and eight-time exposure to OVA alone caused slight to mild goblet cell proliferation in the bronchial epithelium along with very slight infiltration of eosinophils in the submucosa and slight thickening of the subepithelial layer of the airway (Figures 2C, 2G, 3C, and 3G). The four-time co-exposure to ASD and OVA caused moderate to marked goblet cell proliferation (Figure 2D) and eosinophil infiltration and accumulation of lymphocyte (Figure 3D) in the submucosa of airway. The four-time co-exposure caused moderate to marked fibrous thickening beneath the basement membrane in the main bronchus (Figure 2D). All the increases in pathological changes measured after the four-time co-exposure were statistically different (*p* < 0.001) from the 4-contral groups instilled with ASD/OVA alone (Table 1).

**Table 1:** Evaluation of pathological changes in the murine airway

	**Pathological changes**			
Group*	**Proliferation of goblet cells**	**Eosinophils**	**Lymphocytes**	**Thickening of bronchial wall**
4-Control	0	0	0	0
4-ASD	0.31 ± 0.16	0.06 ± 0.06	0.75 ± 0.13^§^	0.75 ± 0.09
4-OVA	0.63 ± 0.18	0.39 ± 0.14	0.94 ± 0.18^‡^	0.63 ± 0.26
4-O+A	3.43 ± 0.17^† ¶ I^	3.14 ± 0.24^† ¶ I^	3.57 ± 0.20^† ¶ I^	3.14 ± 0.26^† ¶ I^
8-Control	0	0	0	0
8-ASD	0.06 ± 0.06	0.06 ± 0.06	1.19 ± 0.19^**^	0.88 ± 0.16 ^‡‡^
8-OVA	0.94 ± 0.29^††^	0.19 ± 0.13	1.38 ± 0.13^**^	0.81 ± 0.16 ^‡‡^
8-O+A	1.81 ± 0.21^** §§ ††† ‡‡‡^	0.88 ± 0.30^†† ¶¶ *** ‡‡‡^	2.19 ± 0.21^** §§ ††† ‡‡‡^	2.00 ± 0.21^** §§ II ‡‡‡^

Data are mean ± SD values.

^*^Four groups of mice were treated intratracheally with normal saline (4-Cont), ASD (4-ASD), ovalbumin (4-OVA) and OVA + ASD (4-O+A) four times at two-week intervals. The other four groups of mice were treated intratracheally with normal saline (8-Cont), ASD (8-ASD), ovalbumin (8-OVA) and OVA + ASD (8-O+A) eight times at two-week intervals. The degree of pathological changes in the airway was estimated: (0) none; (1) slight; (2) mild; (3) moderate; (4) moderate to marked; (5) marked. All values were expressed as mean ± SD (n = 8). Statistical analyses were conducted using Tukey for Pairwise Comparisons.

^†^*p* < 0.001 vs. 4-Control, ^‡^*p* < 0.01 vs. 4-Control, ^§^*p* < 0.05 vs. 4-Control, ^¶^*p* < 0.001 vs. 4-ASD,^I^*p* < 0.001 vs. 4-OVA,^**^p < 0.001 vs. 8-Control, ^††^*p* < 0.01 vs. 8-Control, ^‡‡^*p* < 0.05 vs. 8-Control, ^§§^*p* < 0.001 vs. 8-ASD, ^¶¶^*p* < 0.01 vs. 8-ASD, ^II^*p* < 0.001 vs. 8-OVA, ^†††^*p* < 0.01 vs. 8-OVA, ^***^p < 0.05 vs. 8-OVA, ^‡‡‡^*p* < 0.05 vs. 4-O+A.

The eight-time co-exposure caused a significant increase of goblet cell proliferation compared with the 8-Control (*p* < 0.001) and ASD (*p* < 0.001)/OVA alone (*p* < 0.01); eosinophil infiltration compared with the 8-Control (*p* < 0.01) and ASD (*p* <0.01)/OVA alone (*p* < 0.05); lymphocyte accumulation compared with the 8-Control (*p* < 0.001) and ASD (*p* < 0.01)/OVA alone (*p* < 0.01); and thickening of the subepithelial layer compared with the 8-Control (*p* < 0.001) and ASD (*p* < 0.001)/OVA alone (*p* < 0.001) (Table 1). However, the eight-time co-exposure attenuated goblet cell proliferation (Figure 2G), accumulation of eosinophil and lymphocyte (Figure 3G) in the submucosa of the airway, and fibrous thickening in the main bronchus (Figure 3G) compared with the four-time co-exposure. The eight-time co-exposure caused a significant decrease of proliferation of goblet cells (*p* < 0.05), eosinophil infiltration (*p* < 0.05), lymphocyte accumulation (*p* < 0.05), and thickening of the subepithelial layer (*p* < 0.05) compared with the four-time co-exposure (Table 1).

### Enhancement of cytokines and chemokines in BALF by ASD

**Figure 4:**
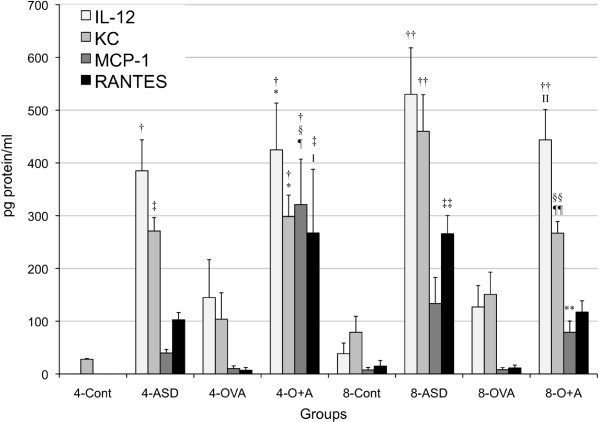
**Expression of IL-12, KC, MCP-1 and RANTES in bronchoalveolar lavage fluid (BALF).** All values were expressed as mean ± SE (n = 8). ^†^*p* < 0.001 *vs* 4-Cont, ^‡^*p* < 0.01 *vs* 4-Cont, ^§^*p* < 0.001 *vs* 4-ASD, ^¶^*p* < 0.001 *vs* 4-OVA, ^I^*p* < 0.01 *vs* 4-OVA, ^*^p < 0.05 *vs* 4-OVA,^††^*p* < 0.001 *vs* 8-Cont, ^‡‡^*p* < 0.01 *vs*. 8-Cont, ^§§^*p* < 0.05 *vs* 8-Cont, ^¶¶^*p* < 0.05 *vs* 8-ASD, ^II^*p* < 0.05 *vs* 8-OVA, ^**^p < 0.001 *vs* 4-O+A.

**Figure 5:**
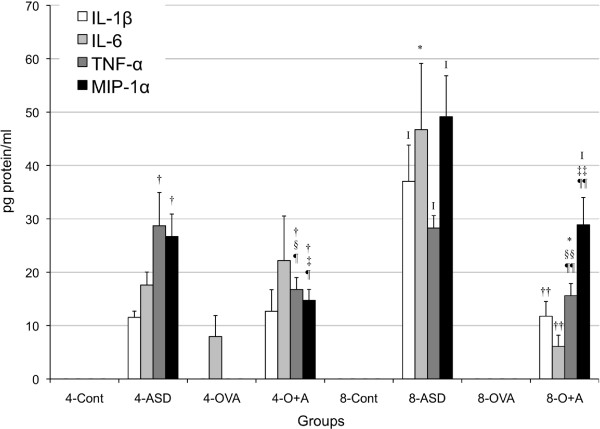
**Expression of IL-1β, IL-6, TNF-α and MIP-1α in bronchoalveolar lavage fluid (BALF).** All values were expressed as mean ± SE (n = 8). ^†^*p* < 0.001 *vs* 4-Cont, ^‡^*p* < 0.001 *vs* 4-ASD, ^§^*p* < 0.05 *vs* 4-ASD, ^¶^*p* < 0.001 *vs* 4-OVA, ^I^*p* < 0.001 *vs* 8-Cont, ^*^p < 0.01 *vs* 8-Cont, ^††^*p* < 0.001 *vs* 8-ASD, ^‡‡^*p* < 0.01 *vs* 8-ASD, ^§§^*p* < 0.05 *vs* 8-ASD, ^¶¶^*p* < 0.05 *vs* 8-OVA.

To investigate the effects of ASD on the expression of cytokines and chemokines caused by OVA, the protein levels of IL-1β, IL-4, IL-5, IL-6, IL-10, IL-12, IL-13, IL-17A, IFN-γ, FGF-2, PDGF-BB, TGF-β1, TNF-α, Eotaxin, KC, MCP-1, MCP-3, MIP-1α, and RANTES in BALF were measured. Both four-time and eight-time exposure to ASD alone increased the expression of IL-12 (*p* < 0.001), TNF-α (*p* < 0.001), KC (four-time, *p* < 0.01; eight-time, *p* < 0.001), and MIP-1α (*p* < 0.001) compared with the each control (Figures 4 and 5). Four -time and eight-time co-exposure to ASD and OVA increased the expression of IL-12 (4- Control, *p* < 0.001; 4- OVA, *p* < 0.05; 8-Control, *p* < 0.001; 8-OVA, *p* < 0.05), TNF-α (4-Control, 4-OVA, *p* < 0.001; 8-Control, *p* < 0.01; 8-OVA, *p* < 0.05), KC (4-Control, *p* < 0.001; 4-OVA, *p* < 0.05; 8-Control, 8-OVA, *p* < 0.05), and MIP-1α (4-Control, 4-OVA, *p* < 0.001; 8-Control, *p* < 0.001; 8-OVA, *p* < 0.05) compared with the each Control and OVA (Figures 4 and 5).

**Figure 6:**
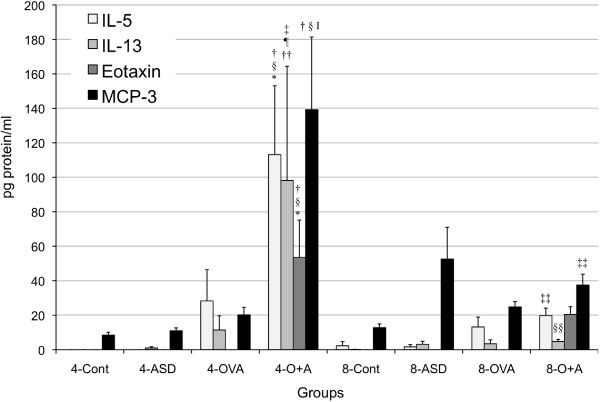
**Expression of IL-5, IL-13, Eotaxin and MPC-3 in bronchoalveolar lavage fluid (BALF).** All values were expressed as mean ± SE (n = 8). ^†^*p* < 0.001 *vs* 4-Cont, ^‡^*p* < 0.05 *vs* 4-Cont, ^§^*p* < 0.001 *vs* 4-ASD, ^¶^*p* < 0.05 *vs* 4-ASD, ^I^*p* < 0.001 *vs* 4-OVA, ^*^p < 0.01 *vs* 4-OVA, ^††^*p* < 0.05 *vs* 4-OVA, ^‡‡^*p* < 0.01 *vs*. 4-O+A, ^§§^*p* < 0.05 *vs* 4-O+A.

Four-time co-exposure to ASD and OVA significantly increased the expression of IL-5 (4-Control, ASD, *p* < 0.001; OVA, *p* < 0.01), IL-13 (*p* < 0.05), Eotaxin (4-Control, ASD, *p* < 0.001; OVA, *p* < 0.01), MCP-1 (*p* < 0.001), and MCP-3 (*p* < 0.001) compared with the 4-Control and ASD/OVA alone. The level of IL-5 (*p* < 0.01), IL-13 (*p* < 0.05), MCP-1 (*p* < 0.001), and MCP-3 (*p* < 0.01) was strongly higher in four-time co-exposure than those in eight-time combined treatment (Figures 4 and 6). The four-time co-exposure increased RANTES expression compared with the 4-Control and OVA alone (*p* < 0.01), whereas eight-time co-exposure to ASD and OVA did not (Figure 4).

**Figure 7:**
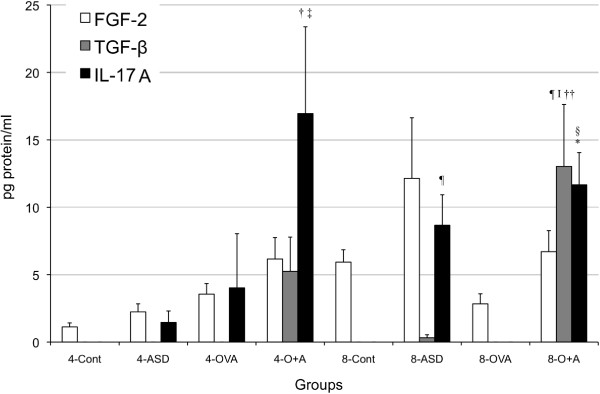
**Expression of FGF, TGF-β1 and IL-17A in bronchoalveolar lavage fluid (BALF).** All values were expressed as mea n± SE (n = 8). ^†^*p* < 0.05 *vs* 4-Cont, ^‡^*p* < 0.05 *vs* 4-ASD, ^§^*p* < 0.001 *vs* 8-Cont, ^¶^*p* < 0.01 *vs* 8-Cont, ^I^*p* < 0.05 *vs* 8-ASD, ^*^p < 0.001 *vs* 8-OVA, ^††^*p* < 0.05 *vs* 8-OVA.

Eight-time exposure to ASD alone increased the expression of IL-1β (*p* < 0.001) and IL-6 (*p* < 0.01) compared with the 8-Control, but the other treatment did not (Figure 5). Only eight-time co-exposure increased the TGF-β1 (*p* < 0.01) expression compared with the Control and ASD/OVA alone (Figure 7). Four-time co-exposure to ASD and OVA increased the expression of IL-17A (4-Control, ASD, *p* < 0.05), and eight-time co-exposure to ASD and OVA increased the expression of IL-17A (8-Control, 8-OVA, *p* < 0.001) (Figure 7). In addition, FGF-2 was not changed and IL-4, IL-10, IFN-γ and PDGF-BB were not detected.

### Enhancement of OVA-specific antibody in serum by ASD

**Figure 8:**
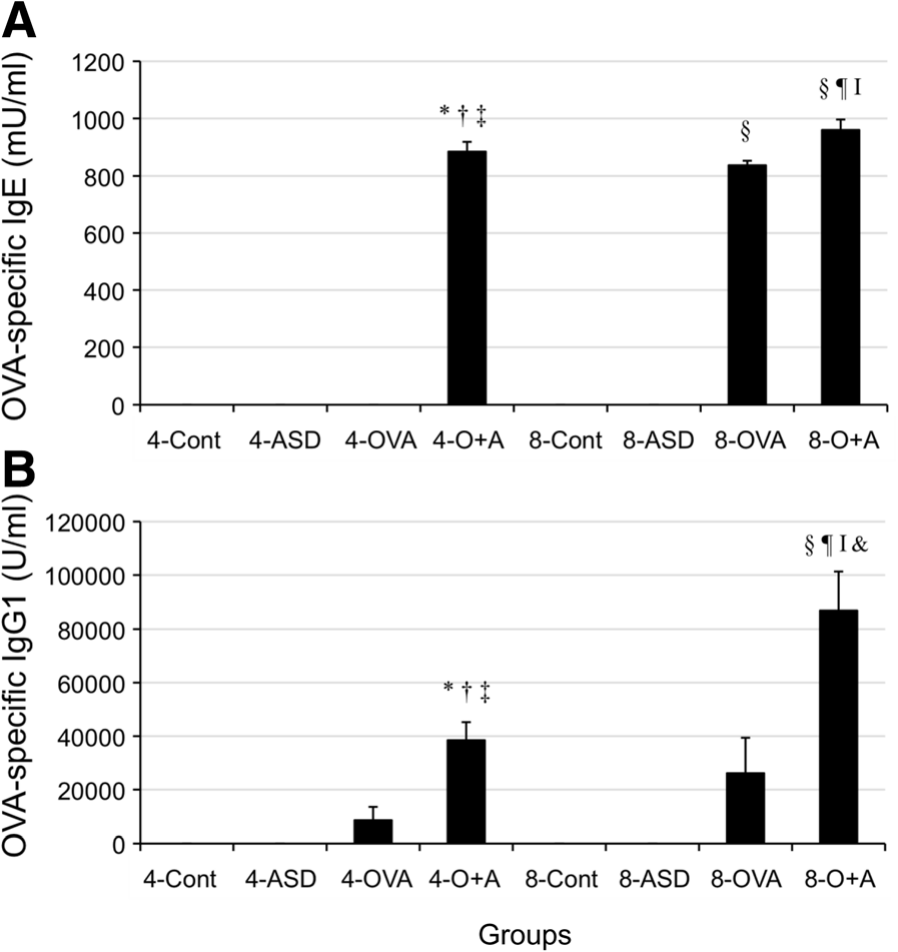
**Effect of ASD on OVA-specific IgE and IgG1 production in serum.** (**A**) shows OVA-specific IgE and (**B**) shows OVA-specific IgG1. According to the manufacturer’s protocol, 1 U of the anti-OVA IgE is defined as 1.3 ng of the antibody, and 1 U of the anti-OVA IgG1 as 160 ng of the antibody. Results are expressed as mean ± *SE*. *p < 0.001 *vs* 4-Control, ^†^*p* < 0.05 *vs* 4-Control, ^‡^*p* < 0.001 *vs* 4-ASD, ^§^*p* < 0.05 *vs* 4-ASD, ^¶^*p* < 0.001 *vs* 4-OVA, ^**^p < 0.001 *vs* 8- Control, ^††^*p* < 0.001 *vs* 8- ASD, ^‡‡^*p* < 0.001 *vs* 8-OVA, ^¶¶^*p* < 0. *vs* 4-O+A, &*p* < 0.05 *vs* 4-O+A.

To confirm the effects of ASD on antigen-induced Ig production, the expression of OVA-specific IgE and IgG1 was performed (Figure 8). As shown in Figure 4, both four-time and eight-time co-exposure to ASD and OVA increased Ig E compared with the each Control and ASD/OVA alone (*p* < 0.001). And the four-time co-exposure to increased IgG1 compared with the 4-Control and ASD alone (*p* < 0.05). Eight-time co-exposure to ASD and OVA increased IgG1 compared with the Control and ASD/OVA alone (*p* < 0.001). In addition, the eight-time co-exposure caused further increase of IgG1 compared with the four-time co-exposure (*p* < 0.001).

## Discussion

ASD events cause deterioration of pulmonary function in asthmatic patients and aggravation of their symptoms [16,17]. Therefore it is important to investigate the series of manifestations in allergic airway disease caused by co-exposure to allergens and ASD for 14 weeks when deciding on a clinical strategy in the treatment of ASD-stimulated allergic airway disease.

The four-time treatment of ASD alone (6-week study) caused bronchitis and alveolitis, and clearly increased neutrophils along with its relevant chemokines MIP-1α, KC (IL-8 in human) and other cytokines IL-12, TNF-α in BALF. These chemokines and cytokines may play an important role in a neutrophilic inflammation processes. The eight-time exposure of ASD alone (14-week study) deteriorated bronchitis and alveolitis, which accompanied with further increase of cytokines IL-1β, IL-6, IL-12, IL-17A and TNF-α; and chemokines KC, MIP-1α, and RANTES in BALF. However, thickening of the subepithelial layer in the airway was slight in both the four-time and the eight-time exposure to ASD alone.

LPS and β-glucan presented in ASD may contribute to cause the neutrophilic inflammation and the production of these cytokines and chemokines, because a previous study reported that LPS and β-glucan induced the expression of their pro-inflammatory molecules [23]. The induction of IL-17A, which was secreted from T-helper 17 (Th17) cells, may be an infection defense reaction to the pathogens such as fungi that adhere to ASD [24].

OVA alone causes a slight proliferation of goblet cells and an infiltration of eosinophils and lymphocytes, and slight thickening of the airway wall, which are the pathology correlatives seen in human asthma. The four-time co-exposure to OVA and ASD enhanced thickening of the subepithelial layer, eosinophil and neutrophils infiltration and the proliferation of goblet cells in the airway, which was evidenced by pathological examination. As an overall trend, these changes and the cellular profile of BALF were paralleled by the expression of Th2-associated effector molecules and eosinophil and neutrophil relevant cytokines/chemokines in BALF as well as the production of OVA-specific IgE and IgG1. It was reported that eosinophils, Th2 lymphocytes and their released inflammatory mediators, such as IL-5 and IL-13, played a crucial role in human allergic asthma [25]. IL-5 has been shown to attract and activate eosinophils, which were implicated in tissue destruction in allergic asthma [26]. IL-13, also released from Th-2 lymphocytes, has been shown to stimulate B cells and to lead to the production of antigen specific antibodies [27] and promote mucous secretion and production of mucous cells, such as goblet cells, in the bronchial epithelium [28]. IL-17A contributes to neutrophil infiltration in the airway inflammation of allergic asthma [24]. Therefore, the airway injury under the co-exposure may be due to enhanced airway inflammation by eosinophilis and neutrophils.

TGF-β1 is well known as a repair and profibrotic cytokine [29]. Hyperplasia of bronchial structural cells, like fibroblasts and smooth muscle cells in the airway, is a typical feature of airway remodeling; hence TGF-β1 plays an important role in the development of airway remodeling [30]. Fibroblast growth factor (FGF)-2 and platelet-derived growth factor (PDGF)-BB also have a significant role in airway remodeling in asthma [31]. However these fibrogenic parameters were found only at low levels or not detected in the four-time co-exposure in spite of the fibrous thickening in the airway. Further increases of these fibrogenic parameters in BALF may be required for serious remodeling of the asthmatic airway to occur.

On the other hand, the eight-time co-exposure to OVA and ASD attenuated fibrous thickening of the subepithelial layer, eosinophil infiltration in the airway as well as eosinophil number and the relevant cytokines IL-5, L-13, and chemokine MCP-3 in BALF compared with the four-time co-exposure group. Oppositely TGF-β1 was significantly increased only in the eight-time co-exposure group.

TGF-β1 is also known to play an important role as an immunosuppressive cytokine [32]. The differentiation of Th1 and Th2 cells is blocked by TGF-β-induced Foxp3^+^Treg cells, which play an important role in immunological tolerance [33,34]. In fact, TGF-β1 can suppress OVA-induced eosinophilic airway inflammation [35]. Over-expression of TGF-β in T cells resulted in the suppression of allergic asthma in a murine asthma model [30]. In contrast, impairment of TGF-β signaling led to increased allergic airway responses in transgenic mouse models compared to wild-type mice [36]. From these reports, we speculate that the attenuation of eosinophil recruitment in the airway under eight-time co-exposure to ASD and OVA may be due to the suppression of Th2 cytokine (IL-13, IL-5) production, which operates by blocking of the differentiation of Th2 cell by TGF-β-induced Foxp^3+^Treg cells. TGF-β induced by the eight-time co-exposure may have an important role in the self-defense reaction for repairing the severe airway injury and for weakening the eosinophilic inflammation enhanced by ASD in an early stage.

Foxp^3+^Treg cells or Type 1 regulatory T (Tr1) cells can suppress the Th2 cell-driven response to allergen through producing IL-10 [37,38], whereas IL-10 was not detected in BALF in this study.

IFN-γ released from Th-1 lymphocytes can suppress Th-2-driven allergic airway responses [39]. However, no increase of IFN-γ was observed in the eight-time co-exposure to OVA and ASD, suggesting that the eight-time sensitization did not cause skewing of the immune response from a Th2 to Th1.

On the other hand, the eight-time combined treatment did not cause the suppression of neutrophil number in BALF compared with the four-time combined treatment. The induction levels of neutrophil relevant cytokines IL-12, IL-17A, TNF-α and chemokines MIP-1α, KC in BALF of the 8-OVA+ASD group were almost similar or higher compared with the 4-OVA+ASD group. The allergic inflammation in the 8-OVA+ASD group may shift to neutrophil-dominant inflammation induced by ASD.

Regarding OVA-specific immunoglobulin production, adjuvant effect of ASD on IgG1 and IgE production was detected in both four-time and eight-time exposure. However, the eight-time exposure could not attenuate the IgE and IgG1 production than those of the four-time exposure. Although a relatively high concentration of Al_2_O_3_ is contained in the ASD, Al_2_O_3_ may not contribute to the adjuvant effect because the adjuvant effect of Al_2_O_3_ particle on their immunoglobulin productions was not detected in our previous study, whereas the possibility of an adjuvant may be in SiO_2_[40]. The augmentation of TGF-β-induced Treg cell differentiation reportedly causes the suppressive effect of IgE production and bronchial hyper responsiveness [41]. From these findings, the suppression of antigen-specific immunoglobulin in serum may require further induction of TGF-β1.

## Conclusions

In conclusion, this study demonstrates that four-time sensitization of OVA with ASD aggravates allergic inflammation along with fibrous thickening of the subepithelial layer in the airway, whereas eight-time sensitization attenuates these changes. These results suggest that eight-time sensitization may cause immune tolerance mediated by TGF-β. The experimental findings in the present study may be useful in considering the best clinical strategy for treating ASD-stimulated allergic airway disease. In future studies, investigation of whether fibrous thickening in the airway remains as irreversible fibrosis after cessation of sensitization of OVA with ASD is called for. The airborne ASD contains biogenic particles, such as bacteria, fungi, virus, pollen, cell debris, and by-product materials derived from air-pollutants. These pollutants may be transported for long distances with mineral particles like silica. The quality and quantity of these components adsorbed on ASD are different based on desert origins and passage routes. Therefore, comparative studies on the respiratory health effects by the ASD with different components are in order.

## Competing interests

The authors declare that they have no competing interests.

## Authors’ contribution

TI designed the research. MH, SY, HT, and MN conducted the experiments. TI and TS analyzed the data and wrote manuscript. TI and GS had primary responsibility for final content. All Authors read and approved the final manuscript.
